# Second-order projection from the posterior lateral line in the early zebrafish brain

**DOI:** 10.1186/1749-8104-1-4

**Published:** 2006-11-29

**Authors:** Ryann M Fame, Carole Brajon, Alain Ghysen

**Affiliations:** 1 Laboratory of Neurogenetics, INSERM E343, Université Montpellier II, place E Bataillon, 34095 Montpellier, France

## Abstract

**Background:**

Mechanosensory information gathered by hair cells of the fish lateral-line system is collected by sensory neurons and sent to the ipsilateral hindbrain. The information is then conveyed to other brain structures through a second-order projection. In the adult, part of the second-order projection extends to the contralateral hindbrain, while another part connects to a midbrain structure, the torus semicircularis.

**Results:**

In this paper we examine the second-order projection from the posterior lateral-line system in late embryonic/early larval zebrafish. At four days after fertilization the synaptic field of the sensory neurons can be accurately targeted, allowing a very reproducible labeling of second-order neurons. We show that second-order projections are highly stereotyped, that they vary according to rhombomeric identity, and that they are almost completely lateralized. We also show that the projections extend not only to the contralateral hindbrain and torus semicircularis but to many other brain centers as well, including gaze- and posture-controlling nuclei in the midbrain, and presumptive thalamic nuclei.

**Conclusion:**

We propose that the extensive connectivity observed in early brain development reveals a basic scaffold common to most vertebrates, from which different subsets are later reinforced in various vertebrate groups. The large repertoire of projection targets provides a promising system to study the genetic encoding of this differential projection capacity.

## Background

The sensory input measured by vertebrate mechanosensory hair cells is hair deflection, yet this input can convey information about a number of different stimuli, such as sound waves, angular acceleration of the head, body movement, or posture. The process by which sensory transduction translates into perception depends on the structure of the sense organ as well as on the distribution of sensory information to specific brain centers through second- (and third-) order projections. Here we examine the second-order projection of a particular set of sensory organs, the mechanosensory organs of the lateral-line system, in the zebrafish embryo.

In amniotic vertebrates, hair cells are restricted to the inner ear where they mediate audition and vestibular proprioceptive functions. In fish and amphibians, mechanosensory hair cells are also present in another sensory system, the lateral line. The lateral-line system is closely related to the inner ear in terms of its placodal origin, projection to the dorsal hindbrain and cytoarchitecture, and was initially thought to underly some sort of auditory function (reviewed in [1,2]). The available evidence suggests, however, that the lateral-line system provides a sense of 'distant touch' that allows fish to perceive their surroundings within a radius of the order of their own body length [3]. This peculiar sense is involved in a large variety of behaviors, ranging from school swimming [4] and the ability to swim against current flow [5,6] to prey detection [7] and/or predator avoidance [8].

The lateral-line system comprises a set of discrete sense organs, the neuromasts, which are distributed on the head and body in species-specific patterns. Individual neuromasts can be either superficial, with the hairs protruding from the epidermis into the surrounding water, or they can be embedded in canals [9,10]. The neuromasts on the head form the anterior lateral-line system (ALL), while those on body and tail form the posterior system (PLL).

In adult fish, PLL sensory neurons have their cell bodies in a cranial ganglion located posterior to the ear and they project ipsilaterally to the 'medial octavo-lateral nucleus' of the hindbrain. This nucleus receives afference not only from the PLL but also from the ALL and from the inner ear. There is segregation of the afference, however, such that the most ventral part of the nucleus receives afference from the inner ear, the medial part from the ALL and the dorsal part from the PLL [11,12].

The second-order projection from the medial octavo-lateral nucleus has been described in the adult of several fish species (reviewed in [12]). It comprises a commissural projection to the contralateral nucleus, where it is presumably involved in the comparison of ipsi- and contralateral inputs, and an ascending projection to a large midbrain nucleus, the torus semicircularis. This projection is bilateral with contralateral predominance. A minor component of the second-order projection extends to the deep layers of another midbrain structure, the optic tectum.

The torus semicircularis is the major target of lateral-line and inner ear information in bony fish. Third-order projections from the torus then convey the information to higher centers, such as the optic tectum, the thalamus and hypothalamus (reviewed in [12]). A midbrain structure homologous to the torus semicircularis is found in all vertebrates, for example, the inferior colliculus of mammals, which is also a major target of auditory information.

Information about lateral-line projections at the end of embryogenesis is much more limited. The first-order projection from the PLL has been described in six-day-old zebrafish larvae [13]. PLL axons bifurcate upon entering the hindbrain and send one branch anteriorly and the other posteriorly, much as in the adult. The projections from the ALL and PLL are separate yet closely apposed, with ALL axons extending ventral to PLL axons [14]. In this paper we examine the second-order projection of the PLL in four-day-old zebrafish. There is no precise boundary between embryonic and larval development in this species. Hatching occurs anytime between two and four days after fertilization, and feeding does not begin until day five, yet organogenesis is complete at two days. Thus, four-day-old fish are at the transition between late embryogenesis and early larval life.

The analysis of second-order projections at this early stage was undertaken for three major reasons. First, early developmental stages could reveal relatively simple patterns of connections that may later be blurred due to subsequent expansion and plasticity. Further, the transparency of the early embryo alleviates the need to reconstruct from serial sections and may, therefore, give a more comprehensive view of the connectivity scaffold. Second, much knowledge has recently accumulated about the genetic control of hindbrain development and regionalization, opening the prospect that the gap between genetic program, brain wiring and behavior may somehow be bridged. Third, examining how and where the information provided by the lateral-line ganglion is processed in the brain may help us understand how this information impinges on behavior and may also give clues about how our own processing of auditory and vestibular information evolved.

## Results

### First-order projection of the posterior lateral line in four-day-old embryos

Axons from the PLL ganglion enter the hindbrain just posterior to the otic vesicle [13]. They bifurcate soon after they enter the hindbrain and form an anterior and a posterior branch. In order to better define the antero-posterior range of the projection, we labeled the PLL sensory neurons in the islet-GFP line, where hindbrain motor neurons provide convenient landmarks for rhombomeres [15]. Specifically, rhombomeres 2 and 3 (r2 and r3) can be identified by the motor nuclei of the trigeminal nerve, r4 by the exit of the facial nerve, r6 by the motor nucleus of the facial nerve, and r7 by a smaller nucleus that comprises the caudal efferent nucleus of the lateral line system [16].

**Figure 1:**
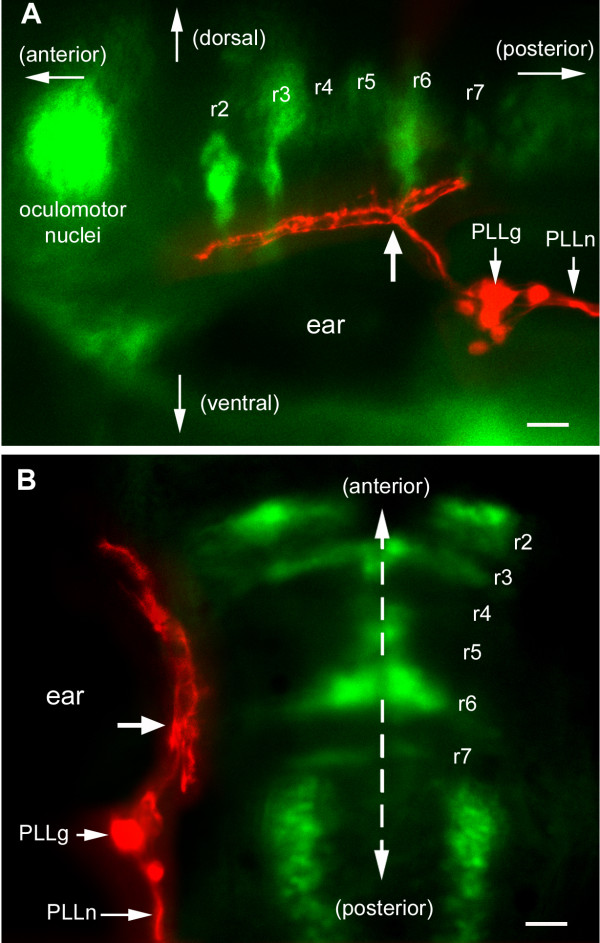
Central projection of PLL sensory neurons in an islet-GFP background, as seen **(a) **laterally and **(b) **dorsally. Rhombomere positions are based on the morphology of the various motor nuclei, particularly trigeminal in r2 and r3, facial in r6, and in r7 the small lateral line caudal efferent nucleus [33]. The exit of the facial nerve in r4, barely visible in (a), provides an additional landmark. DiI has been injected in the PLL nerve (PLLn). The cell bodies of the sensory neurons are gathered in the PLL ganglion (PLLg). Their axons enter the hindbrain posterior to the otic vesicle (ear) and bifurcate at the level of r6 (site of bifurcation marked by a thick arrow in both panels). They extend anteriorly to the level of r1 and posteriorly to the level of r7. The red and green images were taken at different focal planes. In this and in all following figures the dashed line indicates the sagittal plane and the scale bar is 20 microns.

The central projection of the PLL sensory neurons in four-day-old zebrafish is presented Figure 1a (lateral view) and Figure 1b (dorsal view). The hindbrain is not tubular anymore at this stage but is wider anteriorly, due both to anterior expansion of the hindbrain's width and to the presence of the otic vesicle. Combining lateral and dorsal views of several preparations, we conclude that the axons bifurcate at the level of r6 and extend from r1 anteriorly to r7/8 posteriorly, that is, over the entire length of the hindbrain. The width of the projection is rather constant except for a tapering at its anterior and posterior ends.

**Figure 2:**
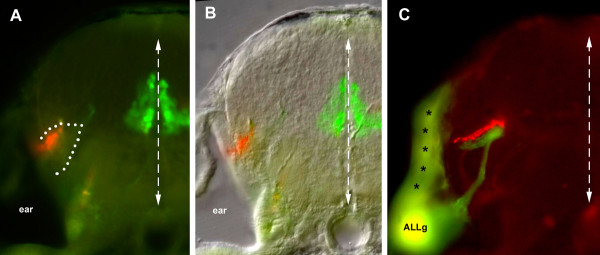
Transversal sections of the afferent projection. At the level of r3–r4, the PLL projection extends at the lateral edge of the hindbrain, while the basal-plate derived motor neurons are present near the midline. Based on **(a) **autofluorescence and **(b) **the Nomarski image, the afferent projection extends within a neuropilic region (dotted line). **(c) **At a slightly more anterior level, the PLL projection labeled with DiI (red) and the ALL projection labeled with DiO (green) are apposed yet segregated. Due to the thickness of the vibratome section (100 micorns), the same section also comprises the ALL ganglion (ALLg) and shows contaminating labeling of the hindbrain surface (asterisks) close to the site of DiO injection.

**Figure 4:**
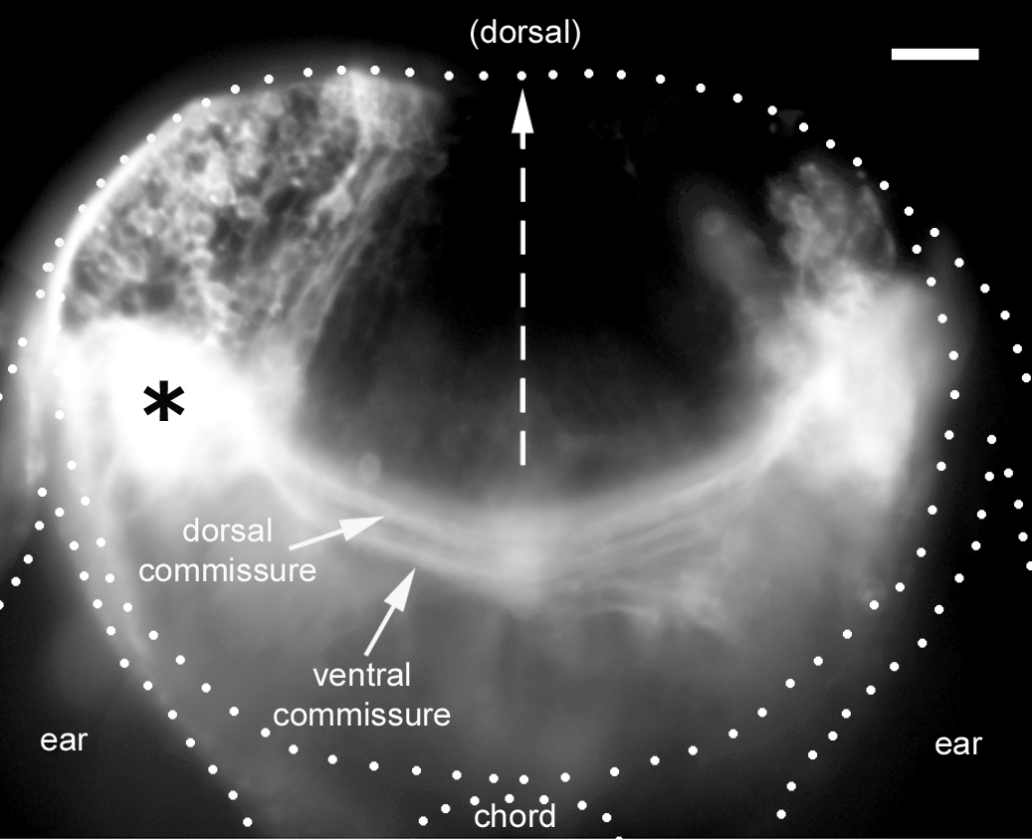
Distribution of second-order neurons. A vibratome section at the level of the site of DiI injection (asterisk) reveals a large number of ipsilateral cell bodies, a more limited number of contralateral cell bodies, and two commissures connecting the left and right lateral line nuclei. The impression of massive spread of DiI is due to the saturation effect of scattered fluorescent light; a lower exposure reveals that the injection was confined to a much smaller region than the white blob in the figure. In this and all subsequent figures, the left PLL synaptic field has been labeled with DiI (in those cases where the injection was on the right, the figures have been inverted to simplify the perception by the reader).

Motor nuclei appear dorsal to the PLL projection in Figure 1a, although the projection extends in the alar plate and the motor nuclei belong to the basal plate. This apparently dorsal position of the motor nuclei is due to the V-shaped structure of the hindbrain, whereby the basal plate becomes largely medial while the alar plate becomes mostly lateral (Figure 2a,b). As previously reported [14], the projection from the ALL extends along the projection from the PLL, just ventral to it (Figure 2c). The two projections are found in a fibrous region (dotted line in Figure 2a) that is itself ventral to the bulk of the alar plate cell bodies (Figures 2a and 4a).

**Figure 3:**
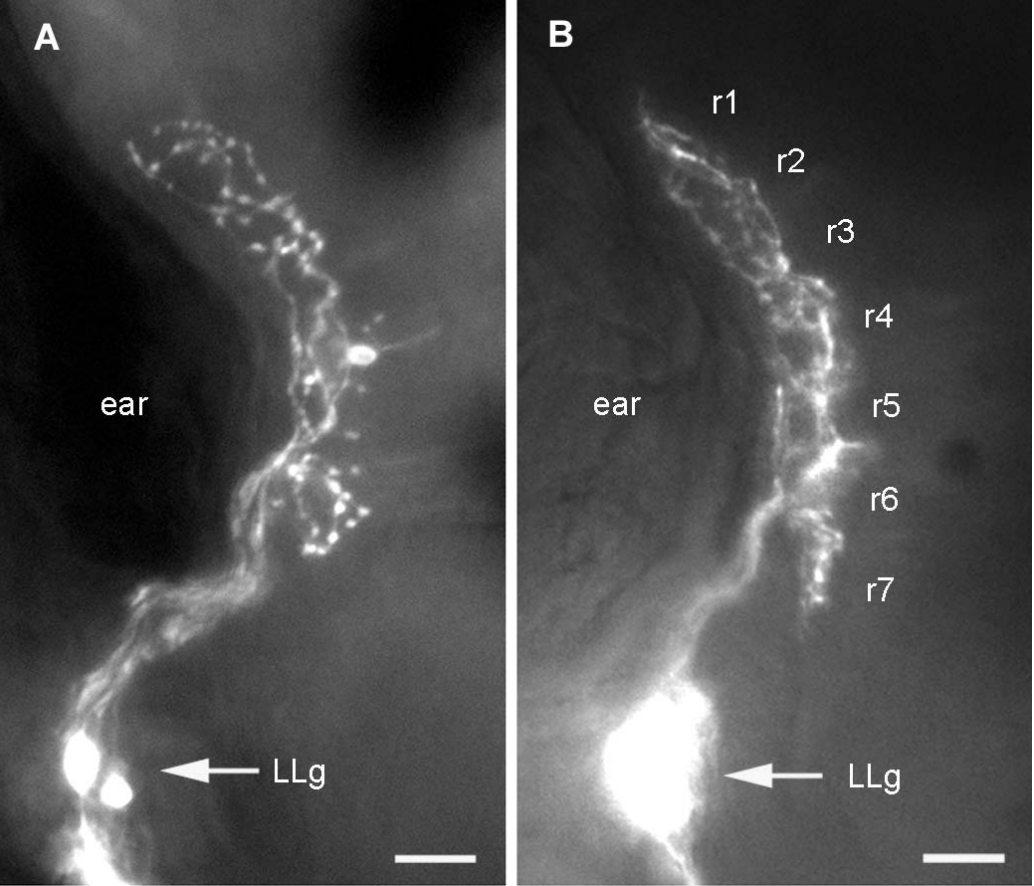
Dorsal aspect of the first-order PLL projection. Dye injection was done either in **(a) **a single neuromast, resulting in the specific labeling of the two neurons that innervate this neuromast or **(b) **in the PLL nerve, resulting in the labeling of many PLL sensory neurons. In both cases the axons outline a well-defined and compact synaptic field. The projection in (b) was observed in an islet-GFP background, allowing us to identify rhombomeres.

When single neuromasts are marked with DiI, two neurons are usually labeled. When seen in dorsal view, the two axons turn out to follow separate courses in the hindbrain and to surround a region where all synaptic boutons are confined (Figure 3a). Both axons and the boutons are essentially in the same plane. The same holds true when the nerve itself is labeled: many more neurons are marked, but their axonal arbours remain as precisely confined as when only one neuromast is labeled (Figure 3b). This feature allows us to precisely define the terminal field of the PLL sensory neurons.

### Second-order neurons

Second-order neurons must necessarily be in direct contact with the terminals of the sensory neurons. In order to specifically label the second order neurons, we first injected DiI in the PLL nerve to label the synaptic field of the afferent neurons. We then injected DiI precisely within this field to label the second-order neurons. Due to the intense brightness generated by the injection it is not possible to examine this region in whole mount embryos, and we relied on thick vibratome sections to examine the distribution of cell bodies and major tracts (Figure 4). Even on sections the intensity of fluorescence makes the region of DiI application appear much larger than it really is (see legend to Figure 4).

The dye injected in the PLL synaptic field labels a large number of ipsilateral neurons located dorsal to the injection site. The cell bodies extend over a large dorso-lateral region, possibly encompassing most of the alar plate (Figure 4). Thus, the segregation between the ALL, PLL and inner-ear regions of the medial octavo-lateral nucleus reported in adult fish has not yet manifested at this early stage. Another clear difference from the adult situation is that the PLL and ALL synaptic fields form adjacent neuropils that are separate from and ventral to the 'nucleus'.

The second-order neurons that pick up the dye injected in the PLL synaptic field extend commissural neurites (presumably axons) to the other side of the hindbrain. Two commissures are labeled, one slightly dorsal to the other. The dorsal one probably corresponds to the inner arcuate fibers described in the adult zebrafish brain and comprises contralaterally projecting fibers. The more ventral one corresponds to the ventral commissure (commissura ventralis rhombencephali) and comprises fibers that project to higher contralateral nuclei ([12] and see below).

**Figure 5:**
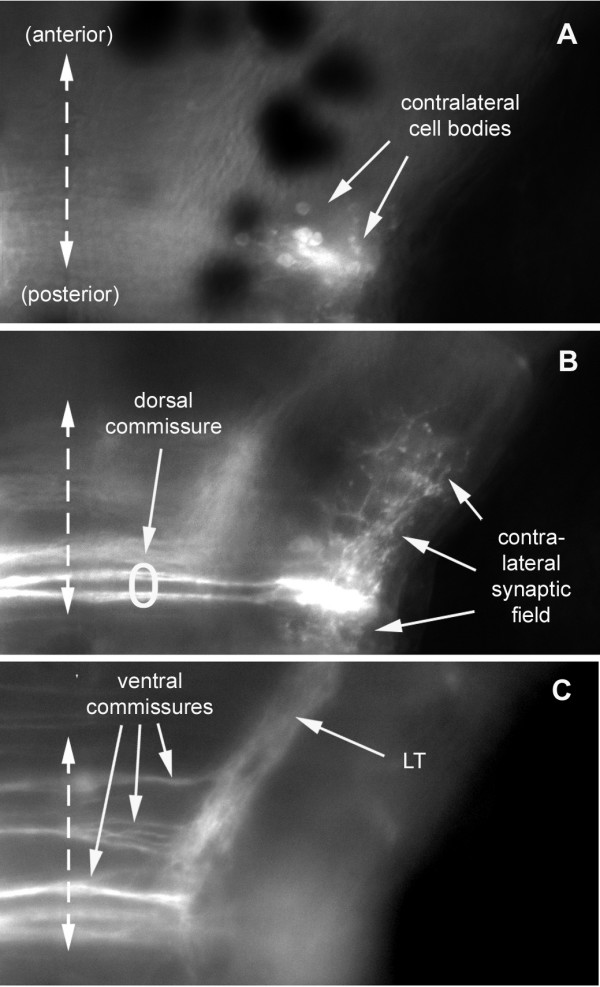
Dorsal aspect of the commissures as seen at three focal levels. From dorsal to ventral: contralateral neurons labeled after injection of DiI in the left PLL synaptic field **(a) **have their cell bodies located at a dorsal level, and **(b) **send their axons along a dorsal commissure and their dendrites invade the contralateral synaptic field. Ipsilateral neurons extending their axon to the contralateral hindbrain nucleus presumably use the dorsal commissure as well (b). **(c) **Other ipsilateral neurons send their axons through more ventral commissures and form the LT branch. All images (a-c) are composites of two consecutive focal planes within an extended Z-series.

In addition to the ipsilateral neurons, a much smaller number of contralateral neurons are also labeled (Figure 4). Contralateral neurons tend to have their cell bodies located laterally (Figures 4 and 5a) and they extend a web of neurites (presumably dendrites) within the contralateral synaptic field (Figure 5b). They send their axons into the labeled synaptic field through a dorsal commissure (Figure 5b), and are probably symmetrical to the second-order neurons that project contralaterally through the dorsal commissure. Indeed, two closely associated dorsal fascicles are seen in Figure 5b, possibly due to simultaneous pioneering of the dorsal commissure from the two sides. Since contralateral neurons take up the dye through their axonal terminals rather than through their dendrites, they are not truly second-order neurons and will be considered together with other back-projecting neurons (see below).

We conclude that there are at least two populations of second-order neurons that extend dendrites into the synaptic field: those that project along the dorsal commissure to the contralateral side, and those that project along the ventral commissure and anteriorly to higher brain centers (Figure 5).

### Second-order projection to higher brain centers: torus and tectum

**Figure 6:**
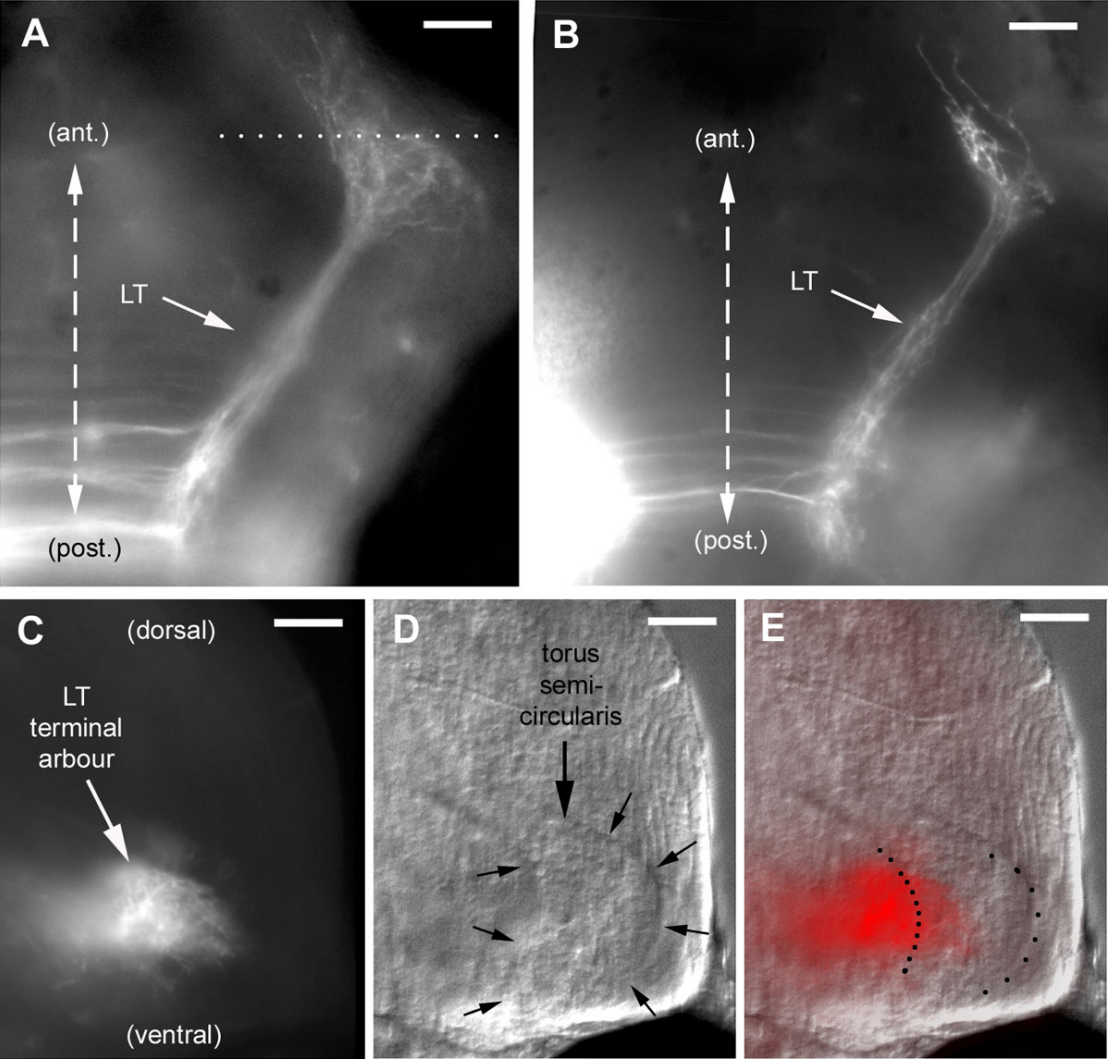
The LT branch extends to the torus semicircularis. **(a, b) **As seen dorsally in whole-mount embryos, the LT projection courses anteriorly and laterally at a level slightly ventral to the ventral commissures, and turns abruptly upon reaching its target (see also Figures 7 and 12). In (a), the dotted line indicates the approximative plane of the vibratome section shown in (c-e). **(c) **In this section, the terminal arbor of the LT projection is seen to extend along the torus semicircularis, **(d) **which is easily delineated under Nomarski optics. **(c, e) **Fine fibers from the LT arbor extend into the torus.

Out of 42 experiments, 38 resulted in the labeling of a well-defined contralateral projection to the midbrain (Figure 6a,b). This projection was named LT (lateral-line to torus) for reasons given below. The fibers form a tight bundle that corresponds to the 'lateral longitudinal fascicle' [17]. The four remaining injections revealed no projection at all except weak commissural fibers for two of them, and a small cluster of posterior neurons for the other two.

In order to identify the target of the LT projection, we performed serial vibratome sections. Fine fibers can be seen to enter the torus semicircularis, one of the few nuclei that are easily observed under Nomarski optics in the early brain (Figure 6d). The bulk of the arborization extends along, rather than within, the nucleus (Figure 6c,e), suggesting that axo-dendritic contact may take place in a neuropil that is adjacent to the nucleus rather than within the nucleus itself. We conclude that the LT branch corresponds to the projection from the lateral-line nucleus to the torus semicircularis previously described in adult forms of other fish and amphibian species. This projection is well developed in zebrafish at four days after fertilization.

**Figure 7:**
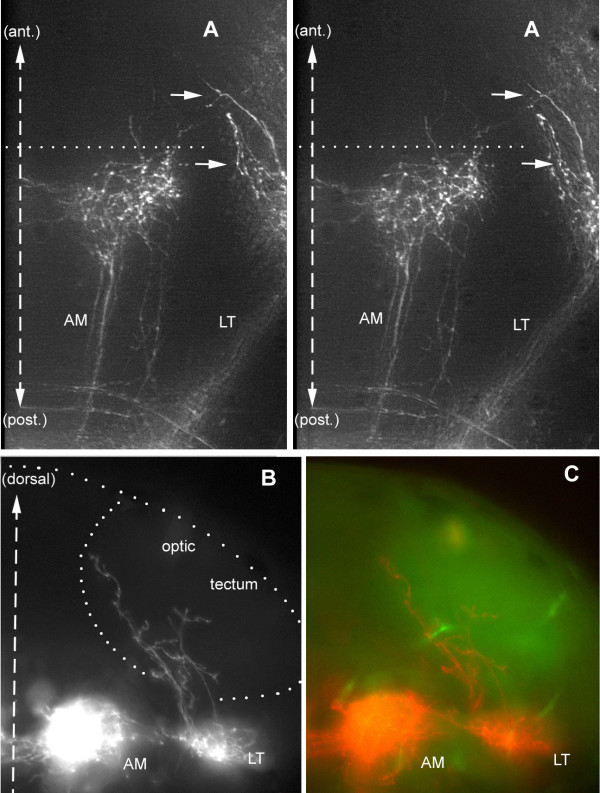
A few fibers escape from the LT projection and climb into the optic tectum. **(a) **Dorsal stereo-view of a whole-mount embryo (to be seen with crossed eyes). The lettering has been adjusted to correspond to the dorso-ventral level of the corresponding features; thus, the arrows that point to the climbing fibers will appear much more dorsal to 'AM', whereas 'LT' will appear slightly ventral to 'AM'. **(b, c) **Climbing fibers seen in a vibratome section. The outline of the tectum in (b) has been drawn based on the autofluorescence pattern seen under blue exciting light (c), which reveals fibrous material (neuropil) as opposed to cell bodies (nuclei).

Out of 25 high-quality LT projections, we found 21 where 1 or 2 fibers escape from the torus and climb dorsally (Figure 7A; the AM projection present in the figure will be discussed below). The four cases where we did not find LT fibers extending dorsally were all cases of posterior injections (r5 or r5/6). As the torus is overlaid by the optic tectum, we assumed these climbing fibers might invade tectal territory. In order to better document this result, we examined sections. Climbing fibers were easily detected in all cases, albeit in very small numbers (two or three fibers). They were observed to extend very widely in the deep layers of the tectum (Figure 7b,c).

Among the 38 projections that showed a contralateral LT branch, only 8 showed some labeling of the ipsilateral LT branch. The labeling was either weak (four cases) or very weak (four other cases). This may be an underestimate, as in one case we observed in sections ipsilateral fibers that we had not detected in the whole mount. It is nevertheless obvious that there is a massive prevalence of contralateral fibers. We did not observe ipsilateral LT fibers climbing to the tectum.

### Specificity of the LT projection

We attempted to evaluate to what extent DiI injected into the PLL synaptic field extends to nearby synaptic fields. The projection from ALL neuromasts is closely apposed to that from PLL neuromasts (Figure 2c). If DiI injected in the PLL synaptic field leaked into the just underlying ALL synaptic field, one would expect to find back-labeling of ALL sensory neurons. This was indeed observed but in only 13 out of 31 preparations; furthermore, 5 of the 13 positives were extremely weak. This indicates that the uptake of DiI is largely or entirely confined to the PLL synaptic field. Thus, we conclude that any branch that we consistently observed very likely arises from neurons that extend neurites within the PLL synaptic field. Such neurons are presumably direct targets of PLL sensory axons (see below for the possibility that some of them would correspond to modulatory innervation from other brain centers).

**Figure 8:**
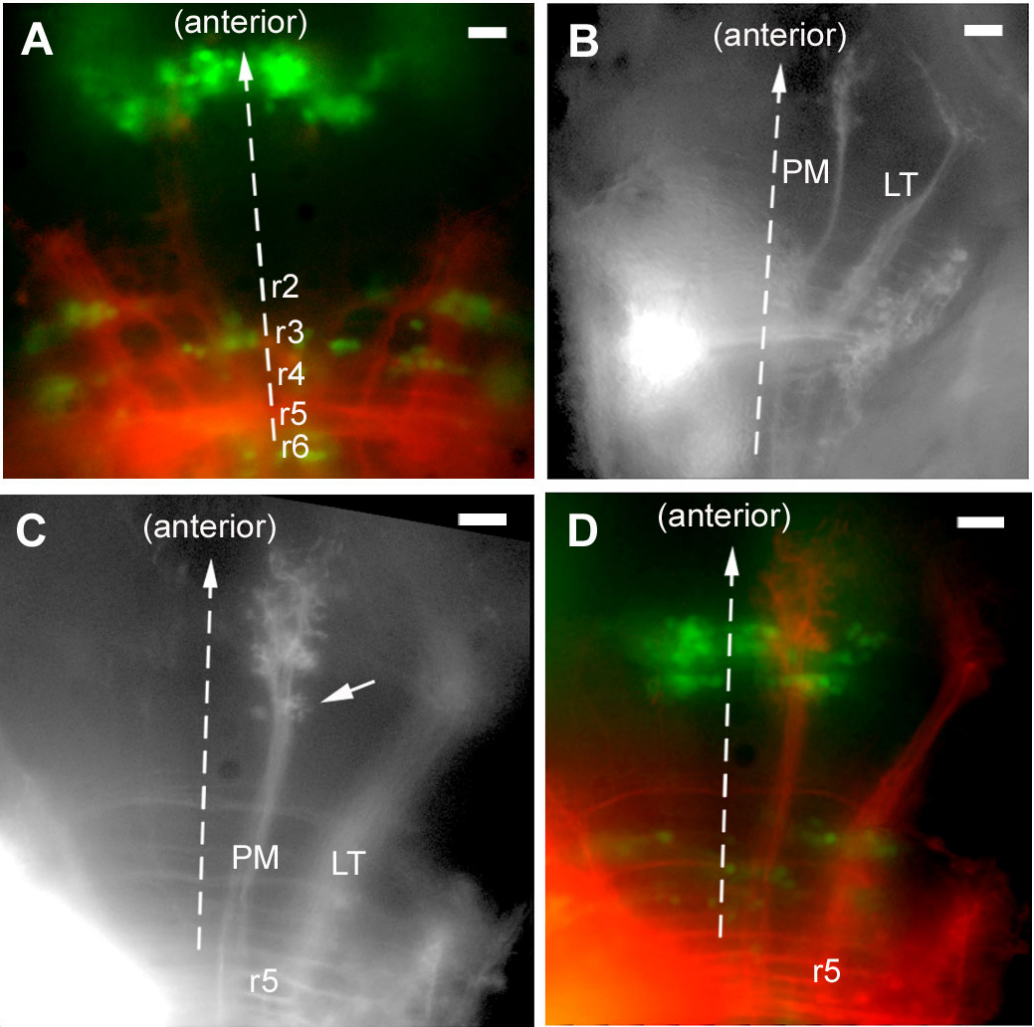
Other posterior projections. **(a) **A projection that was observed in three out of five cases when the site of injection was deliberately out of the PLL synaptic field, slightly medial to it. **(b) **Overall view of the projection resulting from injections in the posterior region of the synaptic field, illustrating the LT projection and posterior medial (PM) projection. **(c, d) **The PM projection extends through the ocular motor complex (oculomotor and trochlear nuclei) and arborizes both posterior (arrow in (c)) and anterior to this cluster.

Since little is known about the second-order projections that extend anteriorly from hindbrain nuclei, it seemed possible that the establishment of a projection to the torus might be a general property of hindbrain neurons. In order to have a quick check for this possibility, we did a small set of labelings where we targeted the injection outside of the primary lateral line projection, more medially, at a distance equal to the width of the projection. In three out of five cases we observed a peculiar bilateral projection that is very different from the LT projection (Figure 8a). We know too little of early brain neuroanatomy to guess what this projection may correspond to, yet the result clearly suggests that the LT projection is not a general feature of all hindbrain neurons.

### Other second-order projections: variations along the antero-posterior axis

In addition to the LT projection, which was consistently labeled in nearly all cases, we also observed other components, some of which were contralateral while others appeared bilaterally symmetrical. The results of a first set of 12 experiments suggested that the pattern of the second-order projection was different when DiI was applied to more anterior or to more posterior regions of the PLL synaptic field. In order to quantify this effect we performed a further set of 31 injections in an islet-GFP background, which allowed us to determine the rhombomeric position where DiI was injected.

The positional identification of the site of injection is somewhat imprecise due to three factors. First, the site of injection is very lateral in the hindbrain, while the rhombomere-specific motor nuclei are more medial (Figure 1b), leading to some uncertainty about the lateral domains of successive rhombomeres. Second, the dorso-ventral level of the projection also differs somewhat from that of the motor nuclei, so that parallax effects may alter the matching. Finally, the position of the motor nuclei relative to rhombomere boundaries has not been precisely defined.

In spite of the uncertainties, however, injection sites can be associated to a given rhombomere or inter-rhombomeric region with reasonable confidence. We estimate that we cannot be wrong by much more than half a rhombomere. This confidence is reinforced by the labeling of commissural axons projecting to the contralateral side. In all cases only one or a few commissures were prominently labeled, corresponding to the site of injection. The rhombomeric identity of commissures can be independently assessed in relation to motor nuclei and in some cases to motor nerves (for example, the facial motor nerve that corresponds to r4). The overall match between the assignments of rhombomeric identities based on the injection site, and those based on the major commissures labeled by DiI, was very good. In 24 cases where commissures could be readily assigned to specific rhombomeres, there were 10 perfect matches between the rhombomeric assignation of injection site and commissures and 14 shifts of half a rhombomere (for example, the injection site being assigned to r2–r3 while the major commissures would sit flatly within r3). In most cases of discrepancy (11 out of 14) the commissures suggest a slightly more anterior rhombomeric position than the one assigned to the site of injection.

**Figure 9:**
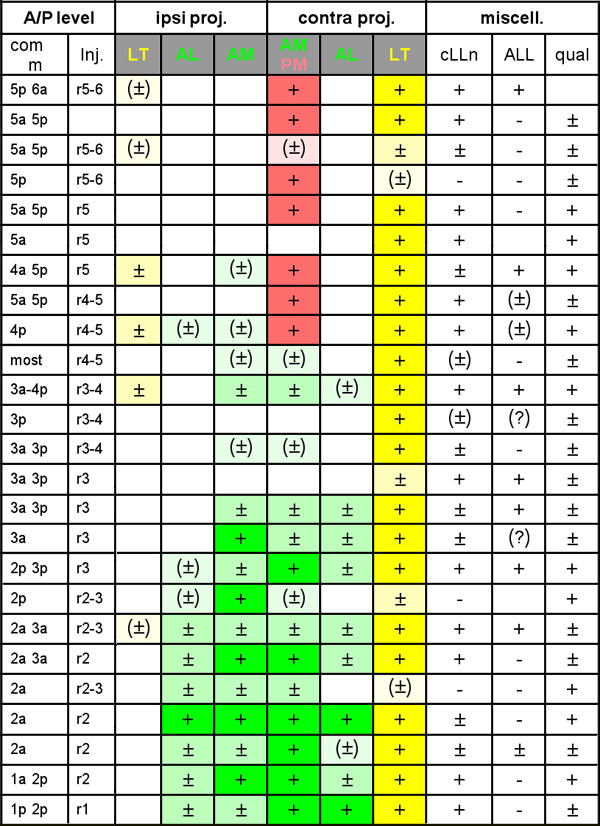
Patterns of second-order projection in islet-GFP embryos. The position of injection was assessed in relation to the rhombomere-specific motor nuclei and was independently evaluated for the position of the commissures (column 1) and of the focus of DiI (column 2). Columns 3 to 5 show the strength of the various ipsilateral branches of the projection; columns 6 to 9 correspond to the contralateral branches. The presence of the various projections has been color-coded; the intensity of the color reflects the strength of the branch: weak, moderate or strong. Note that column 6 corresponds to two overlapping components, one that is typical of the anterior pattern (AM, green) and one that is typical of the posterior pattern (PM, red; see text). Column 9 (cLLn) shows labeling of the contralateral synaptic field and presence of contralateral cell bodies, column 10 (ALL) indicates labeling of nerves of the (ipsilateral) anterior lateral line, and column 11 assesses the overall quality of the preparation. In addition to these 25 preparations, 2 were of low quality but displayed characteristic patterns: LT and PM for an injection in r5 and LT and AL for an injection in r3. Two other preparations did not display any of the components listed in Figure 9 nor did they show commissural labeling, suggesting that the injection site must have been slightly out of the PLL synaptic field.

Figure 9 presents a summary of the data arranged according to the position of the injection. Given that commissures are closer than the sites of injections to the motor nuclei that were used as rhombometic markers, we ordered the cases according to commissural position (column 1). There would be no significant difference if the data were arranged according to the positions assigned to the sites of injection (column 2). Columns 3 to 9 represent the various components that we identified, including the contralateral LT projection that was present in nearly all cases (column 8). The last columns assess the labeling of back-projecting neurons from the contralateral side (column 9), of afferent neurons from the ALL (column 10), and the overall quality of the preparation (column 11). The results demonstrate that the second-order projection is not the same when one injects DiI in the posterior (upper part of Figure 9) or in the anterior (lower part) region of the synaptic field. The same pattern is observed whether the injection is confined to the PLL synaptic field or whether it extends into the ALL synaptic field (column 10), suggesting that, first, the various branches truly belong to the PLL second-order projection, rather than being due to inadvertent labeling of a different system, and second, the second-order projection of the ALL is largely similar to that which we describe for the PLL.

**Figure 10:**
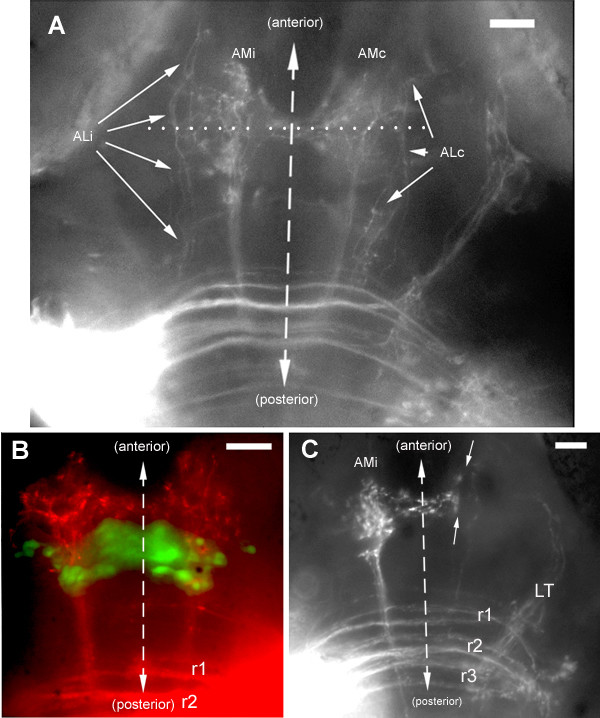
Projections from the anterior PLL synaptic field. **(a) **The ipsi- and contralateral anterior medial projections (AMi, AMc) course through well-defined symmetrical tracts, the MLF (median longitudinal fascicle), and extend very dense commissural fibers. In contrast, the anterior lateral fibers (ALi, ALc) are but loosely fasciculated (arrows). **(b) **The AM projection extends through the ocular motor nuclei and overlaps partly with the PM projection but it differs from the latter in being bilateral with commissural connections, and in extending further anteriorly and laterally. **(c) **The commissural fibers do not invade the contralateral projection but stop sharply at its boundary (arrows)

The projection that corresponds to more posterior injections is characterized by the presence, in addition to the LT branch, of a second contralateral branch (provisionally called PM for 'posterior medial') as shown in Figure 8b. Injections in more anterior regions reveal two bilateral projections, a medial one (AM for 'anterior medial') and a lateral one (AL for 'anterior lateral'), as shown Figure 10a. The contralateral PM and AM branches follow the same tract and their arbours overlap extensively. They were therefore assigned the same column in Figure 9. We will consider them separately, however, because there are clear differences between them, for example, only the AM projection shows bilaterality and commissural fibers. In only 2 out of 25 cases did we observe a projection with mixed features, that is, presence of a strong PM branch typical of the more posterior injections as well as of very weak contralateral AL and AM branches typical of the more anterior injections (Figure 9, lines 8 and 9). We conclude that the two patterns of projections are segregated along the antero-posterior axis of the hindbrain.

Based both on the site of injection and the commissures, we assign the posterior pattern to r5 and r6 and the anterior pattern to r1–r4. Within the r1–r4 domain we observed that AM projections are stronger for more anterior injection sites, but the differences are not large enough to decide with certainty whether there are discontinuities at rhombomeric boundaries or whether there is a continuous trend from r4 to r1. Our impression, however, is that one can distinguish projections corresponding to r1–r2 (AM as strong or stronger than LT) from those that correspond to r3–r4 (AM weaker than LT, ALi barely detectable). Thus, there could be three types of PLL second-order projections corresponding to r1–r2, r3–r4 and r5–r6. An analysis of vestibular pathways in larval frogs has revealed a similar organization where rhombomeres 1 and 2 contribute bilateral ascending projections while rhombomeres 5 and 6 contribute only a contralateral ascending projection [18].

### Second-order projection from posterior levels: the PM branch

The PM branch follows a medial course that corresponds to the medial longitudinal fascicle and arborizes in the midbrain at about the same A/P level as the LT branch (Figure 8b). The PM branch was observed in 12 out of 14 successful injections in the posterior synaptic field, and was, in all cases, strictly contralateral. It often presents a small arborization followed at a more anterior level by a larger arbour with small lateral extensions (Figure 8c). Based on the analysis of sections under Nomarski optics, and on the description of the zebrafish embryonic brain by Mueller and Wulliman [19], we hypothesized that the PM branch might terminate in the vicinity of the ocular motor nuclei (oculomotor and trochlear). To test this hypothesis we examined the relationship of the PM arbour to the midbrain motor nuclei as visualized in the islet-GFP line. The PM branch extends right through the oculomotor-trochlear complex (Figure 8d). The small posterior arbour (Figure 8c, arrowed) lies just posterior to the nuclei and the larger arbour lies anterior to them (Figure 7b). It seems likely, therefore, that the PM projection is mostly concerned with providing lateral line information to the oculomotor system.

In mammals, the medial longitudinal fascicle is involved in the connection between vestibular and oculomotor nuclei, and is responsible for the maintenance of gaze. The PM branch may perform a similar function in fish. The similarity between lateral-line and vestibular second-order projections raises the possibility that the lateral-line system may perform unexplored proprioceptive functions in addition to its better-known role in the analysis of the external water flow. Canal neuromasts would indeed be ideally adapted to provide such information, and the canals themselves, being quite isolated from the surrounding water except for the presence of occasional or terminal pores, may be more akin to the semicircular canals of our inner ear than to water wave detectors.

In most fish species (though not in the zebrafish) there is a prominent canal line running along each flank, so prominent indeed that it is easily seen with the naked eye. Because of their alignment along the antero-posterior axis of the animal, a comparison of the inputs from the left and right PLL canal lines would provide extremely accurate information about angular velocity in the horizontal plane (yaw). This may endow the fish with the capability to build precise memories of their courses in conditions of low ambient light or of no visual landmarks. Interestingly, the blind cavefish *Astyanax *is able to build a spatial map of its surroundings such that it reacts when a particular landmark is displaced relative to other landmarks, even when the distance between two landmarks is such that it cannot possibly perceive both at the same time [20]. These experiments were done in relatively shallow arenas where orientation relies only on yaw and not pitch. Whether the same holds true in the vertical direction, and whether canal lines on the head provide similar information about pitch, is not known.

### Second-order projection from anterior levels: AM branches

The pattern of branches that is associated with the anterior region of the synaptic field is more complex than that associated with the posterior region (Figure 10a). It comprises four bilaterally symmetrical branches: two medial branches (AMi and AMc on the ipsilateral and contralateral sides, respectively) and two lateral branches (ALi and ALc). The intensities of the different branches in different embryos are not always correlated (Figure 9), suggesting that they are formed by different neurons. In most cases, fibers belonging to adjacent projections intermingle in such a way that it is not possible to tell whether a specific fiber emanates, for example, from the ipsilateral ALi or AMi. Likewise the AM branches on the two sides (AMi and AMc) are connected by a very extensive web of commissural neurites, such that it is impossible to tell whether one specific fiber belongs to the the ipsilateral or contralateral component. In spite of these uncertainties, the AM/AL branches present a few robust and interesting features.

The AM branches follow the median longitudinal fascicle much as the PM branch does and their arbour overlaps largely with that of the PM projection. Their arbours extend anteriorly and laterally much beyond the PM branch (Figure 10b), however, making it unlikely that their input would be entirely confined to the ocular motor nuclei. They also differ qualitatively from the PM branch in being bilaterally symmetrical and in extending commissural fibers (Figures 9a and 14). Based on the description of the embryonic brain by Mueller and Wulliman [19], a major target of the AM projection is probably the so-called nucleus of the medial longitudinal fascicle (nMLF) in the diencephalon.

The nMLF provides one of the two major outputs from the brain to the spinal cord (the other being the reticulo-spinal neurons of the hindbrain). Its precise function in the control of body movements is not known yet, though it has been shown that its cells are activated upon gentle tapping of the fish's head, and that it can to some extent substitute for the reticulospinal system in the so-called 'escape reaction' [21]. The lateral line input to this center is highly organized and lateralized (see below), indicating an elaborate processing of lateral line (and presumably other) sensory information. This suggests two levels of motor control by lateral line input: the very fast loop through the reticulospinal neurons, most prominently the Mauthner cells, which mediates the escape response and is, therefore, an emergency response, and a more elaborate but slower processing through the nMLF.

### Lateralization of AM branches

Ipsilateral and contralateral AM branches are almost invariably present together (N = 24), with no discernable lateral prevalence. In 15 out of 24 cases the intensity of the labeling was similar on the two sides of the midline and out of the remaining 9 cases it was stronger on the contralateral side in 5 embryos (and stronger on the ipsilateral side in the other 4, of course).

**Figure 11:**
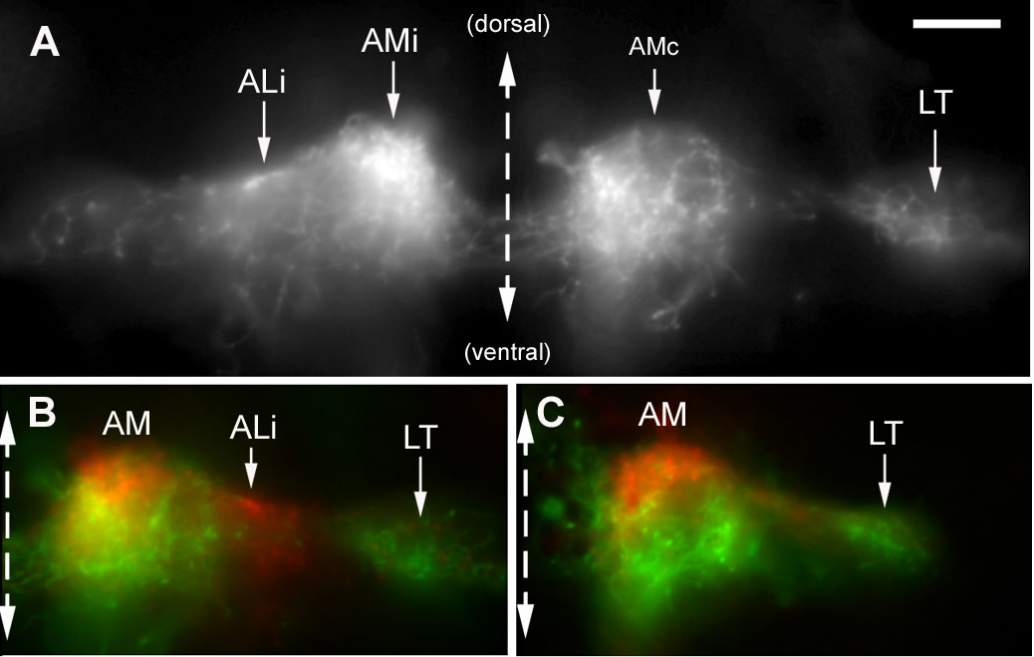
Lateralization of the AM branches. **(a) **Transversal section through the arborization of the AM branches reveals that the ipsilateral projection is very dense in the mediodorsal region of the target area, while the contralateral projection extends more ventrally within the same area. **(b, c) **This difference was emphasized by superimposing the ipsilateral half of the section (colored in red) over the contralateral one (colored in green) for sections of two different embryos. As expected, the LT branch is mostly green, consistent with its contralateral predominance. In (b), the ALi branch happens to be more labeled than ALc and appears, therefore, in red.

Notwithstanding the almost complete correlation between the AM on the two sides, an examination of the best defined projections suggested that the ipsilateral component is slightly more dorsal and extensive than the contralateral component. In order to evaluate this difference we examined sections of several brains. It appears that, even though the general target area is the same on both sides, the distribution of fibers within this area is indeed different on the two sides (Figure 11a). In order to substantiate this impression we superimposed the left side on the right side for four different projections; in all cases we observed an almost complementary organization of the projection on the two sides of the midline (illustrated for two cases in Figure 11b,c).

In one exceptional case we observed a strong labeling of the ipsilateral but not of the contralateral AM (Figure 10c). This embryo revealed that the commissural fibers do not invade the contralateral projections: rather, they stop rather abruptly at the edge, confirming that there is a sharp partitioning of the information from the two sides of the body. In another, less spectacular case, we observed the reciprocal situation where the contralateral AM projection could be seen to send commissural fibers up to, but not within, the ipsilateral AM field.

### AL branches

The AL fibers are more sparse than the AM fibers and do not seem to follow any major tract (Figure 10a). Indeed, they are but loosely fasciculated, contrary to the other components of the second-order projection (LT, PM, AM). They extend lateral and slightly ventral to the AM branches, but they intermingle with the latter in the most anterior part of their projection. Due to this loose fasciculation it is sometimes possible to follow individual fibers. Those few cases show that at least some of the ipsilateral and contralateral AL fibers emanate from different neurons.

**Figure 12:**
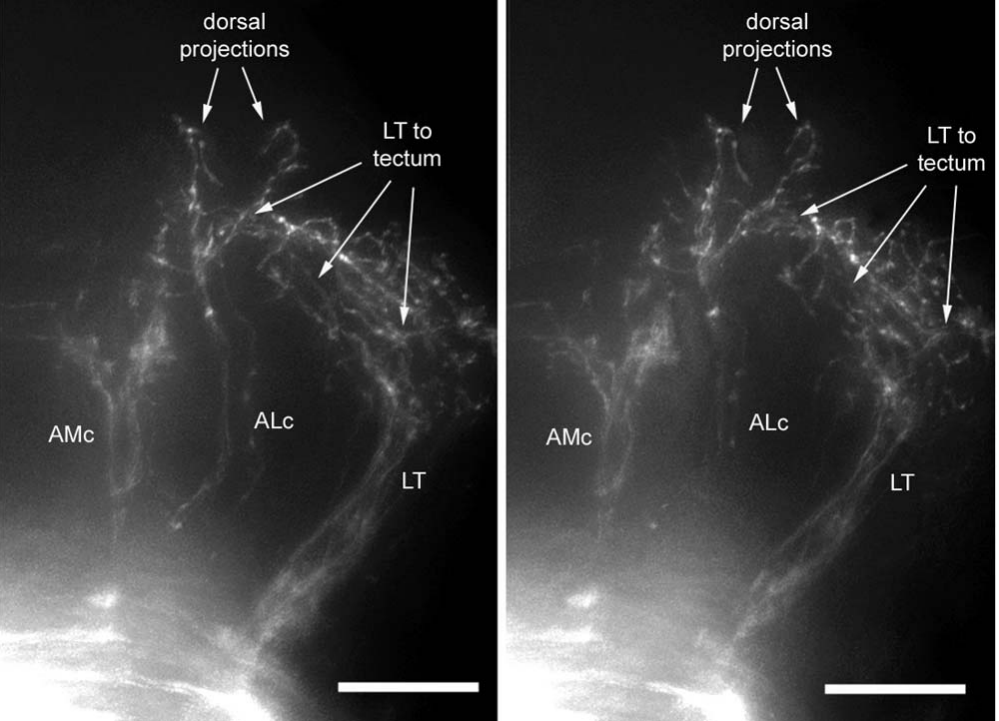
Dorsal extensions of the ALc branch. Stereo-view of a whole mount embryo illustrating the dorsal fibers emanating from the ALc. Lettering emphasizes the dorso-ventral level of the corresponding features, as in Figure 7. When seen with crossed eyes, this view will reveal two fibers climbing from the tip of the ALc projection, slightly anterior to the fibers that extend from the LT projection into the tectum (labeled 'LT to tectum'). The ALc fibers do not reach a level as dorsal as the LT fiber ramification, consistent with a target in the pretectal region of the embryonic brain.

**Figure 13:**
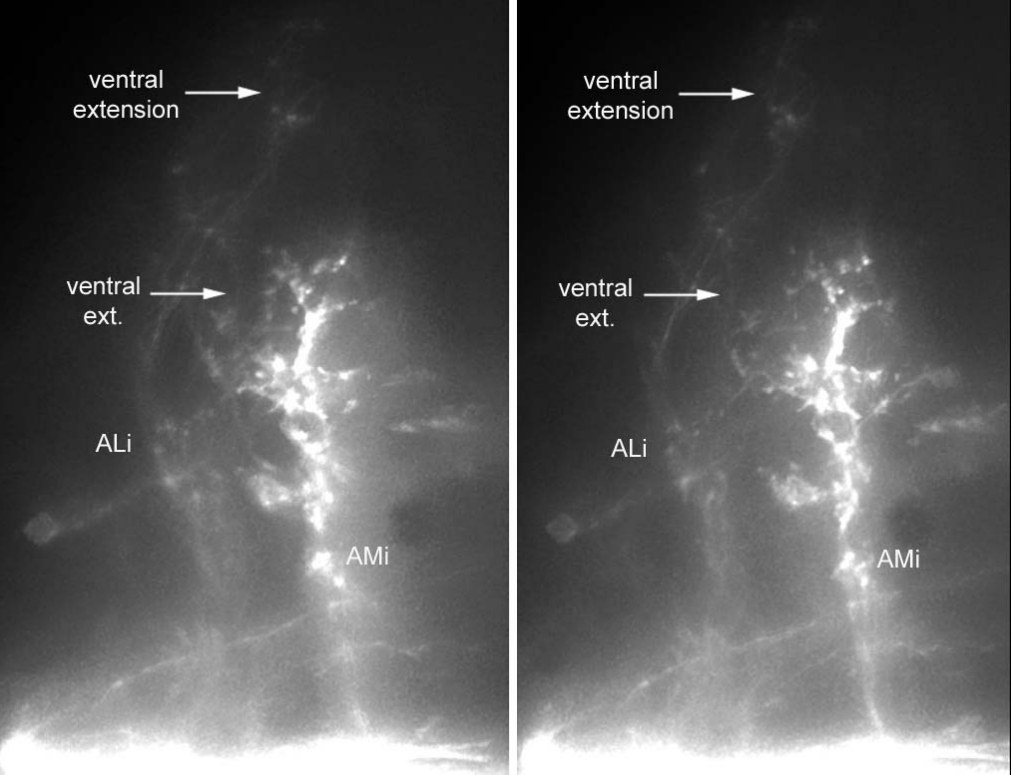
Ventral extension of the ALi branch. Stereo-view illustrating the fibers that extend ventrally from the ALi. One fiber extends ventral to the AMi projection, and more ventral extensions are also observed at more anterior levels.

Some fibers escape from the region where AL and AM fibers intermingle, at the rostral end of the AM projection. Because of the intermingling it is not possible to ascertain whether these fibers emanate from the AL or AM, but our impression is that they are more likely to belong to the AL branch. Some of these escapers extend dorsally (Figure 12), while others extend ventrally (Figure 13) and others escape anteriorly.

**Figure 14:**
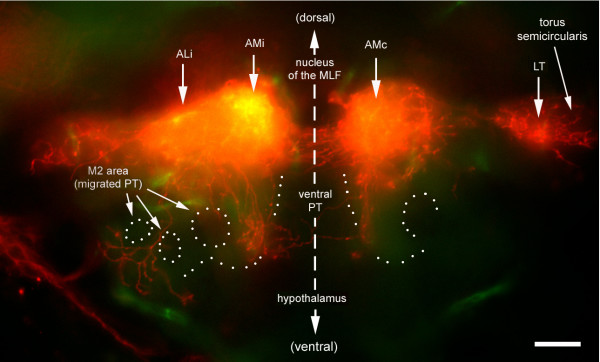
Ventral extension of ALi. Transversal section cut at the level of the commissural fibers (dotted line in Figure 10a). Two prominent ventral extensions are observed. A medial extension is adjacent to (and crosses) the posterior tuberculum (PT), a thalamic derivative. A more lateral extension arborizes in the M2 area. This region will form the preglomerular nuclei, the major diencephalic target of third-order lateral line projection in the adult. The outline of the major nuclei (dotted line) is based on autofluorescence, which reveals fibrous material (neuropils) as opposed to cell bodies (nuclei).

The dorsally directed fibers terminate in a region that is anterior to the tectum (Figure 12), most probably the pretectum [19]. A detailed analysis of high-quality preparations revealed that dorsally directed fibers are very often associated with the contralateral AL (in 11 out of 16 preparations) but not with the ipsilateral AL (1 doubtful case out of 13 preparations).

In contrast, the ventrally directed fibers are associated with the ipsilateral AL (or AM) in 8 out of 13 good preparations, but never with the contralateral AL or AM. Due to optical distortions, this region is less easy to visualize properly in whole mount preparations than the midbrain and hindbrain, and we relied on vibratome sections both to confirm the asymmetry between ipsilateral and contralateral ventral projection and to identify the target region. This revealed the presence of at least two well-defined ventral branches of the ALi (Figure 14).

One branch extends ventrally and laterally and arborizes extensively near the so-called 'migrated portion of the posterior tuberculum' (M2). This region will give rise to the preglomerular nuclei in the adult brain [19]. Interestingly, the lateral preglomerular nucleus is the major adult target of the third-order projection sent out by the torus semicircularis. A second branch extends ventrally and more medially, and a few fibers cross the midline to the contralateral side. This branch is closely apposed to the major diencephalic nucleus of the fish brain, the posterior tuberculum.

**Figure 15:**
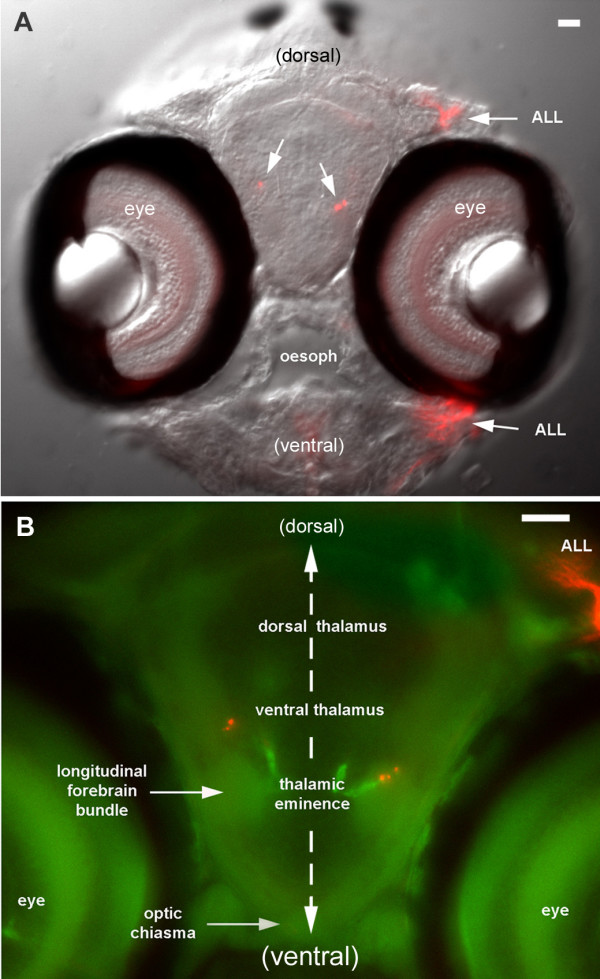
Anterior extensions of ALc and ALi. **(a) **Transverse section of the head at the level of the optic chiasma. The injection in the PLL synaptic field extended to the ALL synaptic field such that ALL neuromasts and nerves are also labeled (ALL). **(b) **Position of the labeled fibers in relation to the major forebrain subdivisions, based on the autofluorescence pattern (green). Since the thick section is subjectively seen from the front, the injection site appears on the right side of this figure (left side of the fish).

Finally, the anteriorly directed fibers also seem to follow slightly different courses on the two sides as seen in whole mount preparations. This impression was confirmed by examining cross-sections at the level of the optic chiasma (Figure 15). The ipsilateral branch courses along the thalamic eminence, a region that is thought to give rise to the entopeduncular nucleus located at the junction between diencephalon and telencephalon [19]. Intriguingly, the posterior entopeduncular nucleus is, in amphibians, the second major target of the ascending projection sent by the lateral line region of the torus semicircularis [22]. Unfortunately, little is known about the connectivity of this nucleus in adult fish. The contralateral fibers course at a more dorsal level that probably corresponds to the ventral thalamus, another target of the ascending projection from the torus semicircularis in amphibians [22].

### Back-projections from higher brain regions

In addition to the second-order neurons that collect the information coming from the PLL axons, injections in the PLL synaptic field should also label neurons from other brain centers that send their axons back to, or very close to, the PLL synaptic field. (for example, to modulate its activity or contribute to signal processing). As mentioned above, we often observed contralateral cell bodies that most likely correspond to back-projecting (or reciprocally projecting) hindbrain neurons. Contralateral cell bodies were readily detected in 21 out of 40 good preparations. They extend dendrites over the entire extent of the contralateral PLL synaptic field. Contralateral neurons were observed after injection at any antero-posterior level, and they always sent their axons within the corresponding commissure(s), suggesting that this type of neuron is present at all antero-posterior positions.

Since the contralateral neurons are probably symmetrical to the ipsilateral second order neurons projecting contralaterally, one might expect to have a similar labeling of both neuronal populations, yet contralateral cell bodies were observed in only about half of the preparations (21/40). One straightforward explanation could be that the axons from contralateral neurons do not terminate within the sensory synaptic field but at some short distance, such that they are less likely to take up the dye. In support of this explanation, we observed that the number of contralateral cell bodies correlates well with the density of neurites in the contralateral synaptic field, but not with the number of ipsilateral cell bodies or intensity of ipsilateral labeling (Figure 9, column 9).

We did not observe cell bodies in more anterior regions of the brain, yet it seems likely that there is some feedback from higher brain regions onto the PLL nucleus. Since hindbrain contralateral neurons seem to project near to but not within the PLL synaptic field, we thought that axons from higher brain centers may be similarly confined to a region some distance away from the PLL synaptic field. In a few cases we injected larger quantities of DiI to increase the probability of labeling neurons that would back-project near to, but not within, the PLL synaptic field.

**Figure 16:**
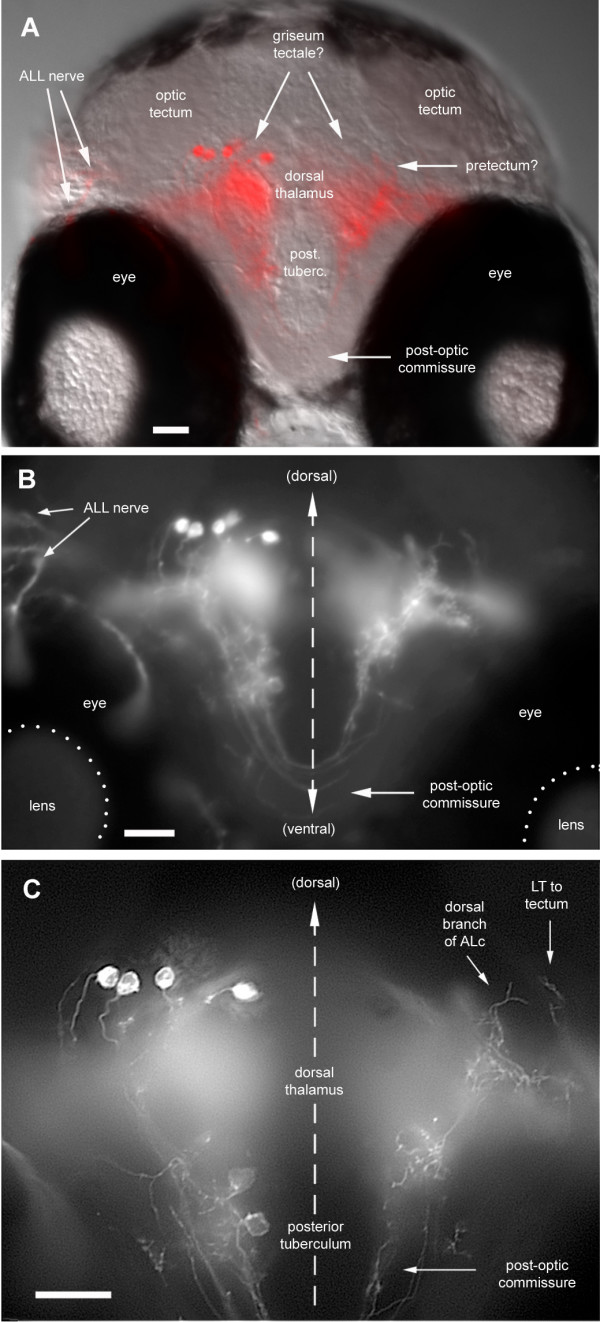
Anterior neurons projecting back to the hindbrain. **(a) **Transverse section revealing the overall pattern at the level of the postotic commissure. A large amount of DiI was injected, resulting in the back-labeling of ALL neurons on the ipsilateral side as well as of central neurons at the midbrain-forebrain boundary. **(b) **Higher magnification revealing fibers that follow the post-optic commissure. Since such fibers are only observed when back-projecting neurons are labeled, we believe that they belong to the latter. **(c) **Detail of the same preparation cleaned with the 'Rapid Deconvolution' program of IPLab (see Materials and methods) to enhance the cell bodies of the back-projecting neurons. Also present on the contralateral side of this section is a fiber of ALc extending dorsally, possibly into the pretectum, as well as the anteriormost branchlet of a LT fiber that extends into the optic tectum.

Large injections of DiI did not result in the labeling of new types of projections, nor in more complex patterns: we recognized the same projections that were documented in the previous sections and they still segregate according to the anterior or posterior site of injection in the primary synaptic field. We did, however, observe labeling of anterior cell bodies (Figure 16). Two types of neurons were labeled: some have their cell bodies in a very anterior and dorsal position (Figure 16a,b). Based on the position of the dorsal AL extensions on the contralateral side, this region may correspond to the hypothetical 'griseum tectale' of Mueller and Wullimann [19]. A second population of back-projecting neurons has their cell bodies close to the postotic commissure and sends neurites across the commissure (Figure 16c). Both types were strictly ipsilateral, and always in limited numbers.

## Discussion

**Figure 17:**
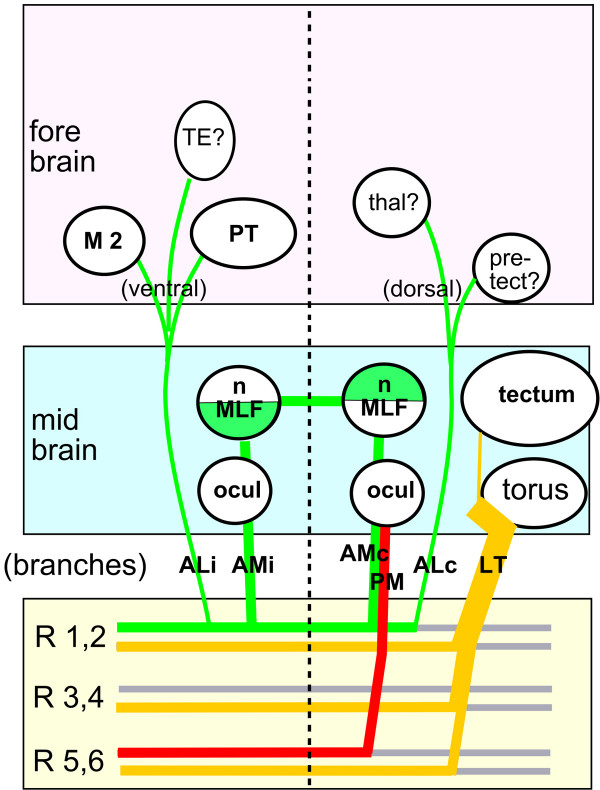
Summary of the various branches described in this paper and of their putative origins and targets. Contralateral projections within the hindbrain have been omitted for clarity. The various projections have been color-coded as in Figure 9. Uncertainties over the target regions are described in the results. M2: migrated portion of the posterior tuberculum (will give rise to the preglomerular nucleus, a major target of the third-order pLL projection in fish). PT: posterior tuberculum, the major diencephalic center in fish. TE: thalamic eminence, the putative origin of the entopeduncular complex (a major target of third order PLL projection in amphibians). Rhombomeres r3 and r4 are shown to send only a LT (yellow) branch; the data presented in Figure 9 suggest that they also send AM and/or AL (green) branches but to a minor extent.

Discussion and interpretation of our observations has been included in the previous sections, and Figure 17 summarizes our results. Here we want to concentrate on four unexpected aspects of our results: first, the surprising richness of the early connectivity in the embryonic brain, compared to that previously reported for adult brains; second, the organization of nuclei and neuropils; third, the lateral asymmetry of the PLL second-order projection; and fourth, the functional significance of the connections that we have identified.

### Exuberance?

The richness of early connectivity that we report in this paper might give an impression of exuberance [23]. Exuberance is thought of as an early, immature developmental stage of uncontrolled neuritic growth. This exuberance is assumed to be called to order sooner or later through processes related to neural activity, such that many fewer connections are observed in the adult than at early developmental stages.

In the case of the PLL, the late embryo reveals many more second-order ascending projections than have been reported in the adult of other fish species examined so far, supporting the general idea of early 'exuberance'. Yet the projections that we observe are extremely reproducible and well-defined. It is crucial, therefore, to know what the targets of this early 'exuberant' stage are. There are, unfortunately, obvious limitations to our identification of target areas. Brain nuclei are not well individualized in four-day-old fish (with a few exceptions such as the torus semicircularis). For example, it has been observed that tyrosine hydroxylase producing cells of the ventral thalamus and posterior tuberculum appear contiguous in five-day-old embryos, even though they are spatially well separated in adult brain [24]. Thus we have mostly relied on the tentative assignation of major brain subdivisions proposed by Mueller and Wullimann in their atlas of the early zebrafish brain [19].

The putative targets that we could assign to the various branches of the second-order projection correspond surprisingly well to known adult targets of the third-order projectionthat arises from the lateral-line region of the torus semicircularis. This includes the deep layers of the optic tectum (Figure 7) and the future preglomerular nucleus (Figure 14). We also find thalamic targets that do not seem to be used in adult fish but have been documented as targets of the torus semicircularis in amphibians (putative entopeduncular nucleus and ventral thalamus; Figure 15).

We find branches to the oculomotor nuclei that resemble the ascending vestibular projection common to all vertebrates. Although we cannot rule out that this part of the projection is a contamination that actually belongs to the vestibular and not to the PLL projection, we think that this explanation is very unlikely. The first-order projection from the inner ear extends ventral to the projection from the ALL, which itself extends ventral to that of the PLL [12]. Since the ALL was retrogradely labeled in less than a third of the cases, it is difficult to imagine that the dye would have spread to the inner ear projection in all cases. Yet we observed branches extending to the oculomotor nuclei in all cases where we injected in r2 and r3, making us confident that this part of the projection truly corresponds to the PLL. In addition, we observed that the projection to the oculomotor nuclei further extends to the region of the nMLF, as reported in mammals but not in adult fish.

The extensive connectivity that we observe in four-day-old fish may be interpreted in two ways. One possibility is that there is indeed a substantial amount of uncontrolled exuberance, and that the difficulty in defining prospective nuclei at this early stage makes it all too easy to find homologies where there are none. The other, more attractive possibility is that the embryonic brain reveals a primitive, general-purpose connective scaffold or blueprint that has been strongly conserved among vertebrates and from which subsets of connections may have been selected for further development in different families. This would be similar to the remarkable conservation of pioneering central nervous system connectivity [25] or of the embryonic peripheral nervous system pattern [26] in widely divergent insects, even though the conserved patterns are modulated and used for very different purposes later in development.

### Organization of the early brain: nuclei and neuropils

We observed in several cases that projecting fibers extend to neuropilic regions rather than into nuclei. The separation between nuclei and neuropils is apparent in the hindbrain already, as the fibers from the anterior and posterior lateral line extend ventral to the medial octavo-lateral nucleus. The second-order LT fibers arborize along rather than into the torus semicircularis, and the PM fibers extend around the oculomotor nuclei. This is in contrast to the situation in adults, where projecting fibers usually end up within their target nuclei.

The change from larval to adult patterns could be due to a progressive extension of the projecting fibers into the nuclei, as could be the case with the LT projection (Figure 6). Alternatively, one could imagine that, after the second-order neurons have extended their dendrites into the synaptic field, their nuclei translocate to end up within this field. A similar process of nuclear translocation has been demonstrated to account for the major morphological difference between arthropod and vertebrate motor neurons [27]. This would also explain how the second-order neurons of the PLL are interspersed with other neurons (presumably ALL and inner ear second-order neurons) in the early brain, yet become segregated in distinct regions of the medial octavolateral nucleus in the adult.

Our data also suggest that there is a substantial level of organization of the sensory neuropil in the hindbrain of four-day-old fish. The afferent projections of the ALL and PLL are apposed yet clearly segregated (Figure 2c) [14]. The contralateral (reciprocal) second-order neurons do not project within the PLL synaptic field but at some close distance. The same is true for diencephalic neurons that send descending projections to the hindbrain: their axons seem to terminate further away from the PLL synaptic field, since they can be labeled only with more massive injections of dye. Connectivity is usually described in terms of nuclei, and not in terms of synaptic fields (for example, [22]). There is no doubt that injecting dyes within nuclei is a useful way to study the overall connectivity of the brain. Our data suggest, however, that this approach may not be optimal to follow the flow of information. Injections within synaptic fields may provide more accurate information about this flow, at least at early developmental stages where the segregation between neuropils and nuclei appears more prevalent than in the adult.

### Lateralization

All levels of the lateral line projection are massively lateralized. The primary projection is entirely ipsilateral, while the second-order projection to the torus semicircularis is almost entirely contralateral. Thus, the information from the lateral line will end up mostly in the contralateral torus. The visual information goes directly to the contralateral tectum. Visual and lateral-line information will, therefore, end up in adjacent regions of the contralateral midbrain.

The tectum is one of the major integrative centers of the fish brain, one that is 'exquisitely designed for integrative orientation tasks' [17]. It is, therefore, not surprising that its sensory input should correspond to the same half of the animal (be it ipsilateral or contralateral). Lateralization of the brain may then affect coordinately various aspects of behavior. For example, it is known that the exploratory visual behavior of fish relies mostly on the right eye, that is, on information arriving on the left tectum [28]. In the blind cavefish *Astyanax*, exploratory behavior most likely relies on lateral line information. In this species, exploratory behavior involves exposing the right side of the fish to new stimuli or objects [29], such that the relevant information is again sent to the left midbrain.

In contrast to the prominent lateralization of the LT projection, the anterior branches seem more bilaterally balanced. This resembles the situation in frogs, where the projection from vestibular to oculomotor nuclei is bilateral for rhombomeres 1 and 2, and contralateral for rhombomeres 5 and 6 [18]. Upon closer examination, however, we observed that all components of the second order projection differ between the ipsi- and contralateral sides. The AM branches follow similar courses on the two sides of the brain, and their major target is the same on either side, but the distribution of second-order axons within this area is almost complementary between the two sides. Likewise, the AL branches are, to a large extent, symmetrical, yet the ipsilateral one projects ventrally to components of the diencephalic posterior tuberculum, while the contralateral ones project dorsally to the pretectum (or a nearby region). Finally, the fibers that extend anteriorly also follow parallel courses on the two sides of the brain but the ipsilateral one courses along the thalamic eminence, while the contralateral one follows a slightly more dorsal course along the ventral thalamus.

The central connectivity of fish is often described as largely bilateral. Lateral prevalence is limited and variable from one species to another. Our observations do not fit easily with this view. Not only do we observe almost complete lateralization in the second-order projection of the lateral-line system, but we also reported previously an almost complete lateralization of the efferent control of the lateral line organs [30]. The discrepancy could be due to at least two reasons. First, zebrafish might be unusually lateralized; second, embryonic connectivity might be unusually lateralized. The first explanation sounds somewhat unlikely but cannot be dismissed altogether. The second explanation would imply that, far from being exuberant, the connectivity of the embryonic central nervous system is, on the contrary, very tightly controlled. The variable bilaterality of the adult connections could then be a later addition to a stereotyped, completely lateralized scaffold of projections. Interestingly, it has been reported that there is a large increase in bilaterality of the vestibulo-ocular projection between larval and juvenile turbot [31]. This increase has been correlated to eye migration during metamorphosis of the flatfish [31], but our observations of the early zebrafish brain suggest that increased bilaterality may be a general feature of post-embryonic development.

### Function

The analysis of PLL connectivity is complicated by the unexpected segregation of nuclei and neuropils, such that the projection fibers do not end up within nuclei, but in their vicinity. In the case of the torus semicircularis this poses no real problem since a small part of the arborization is seen to enter this nucleus (Figure 6), and there are abundant data showing that the torus is indeed the major target of second-order PLL projection. The situation is more complex in other regions, since we have no way to decide from our data which of the adjacent nuclei is the target of projecting fibers.

Given the many restrictions to the analysis of the data, we will nevertheless propose some general interpretation of our results, progressing from posterior to anterior levels. The reciprocal connection between the left and right synaptic fields most likely serves to compare the information from the left and right sides of the body, a computation that may be useful whenever a fast comparison between left and right inputs is essential (such as in rheotaxis or in the escape response). It seems likely in this context that the contralateral input would be inhibitory and would specifically impinge on the subset of second-order neurons that are involved in this comparison.

The projection to the torus semicircularis has been the object of much analysis, in particular the extent of segregation between lateral-line and inner ear projections, and will not be discussed further. Suffice to say that this projection is a robust aspect of vertebrate midbrain connectivity and plays an important role in the processing of auditory information in tetrapods. The existence of a few branches of the LT projection that extend directly to the deep layers of the tectum is interesting, since these fibers provide an early connection between lateral line and visual centers. As such they may play an important role as pioneers for the later, massive connection between torus and tectum. Alternatively, but not exclusively, such an early input may play a role in the patterning of the deep layers of the tectum, thereby ensuring the proper targeting of later fibers connecting torus to the tectum.

The projection to the eye motor nuclei and to the nMLF has not been reported previously. Given the prevalence of this component in the vestibular projections in mammals, its importance in the zebrafish embryo suggests that the lateral line system may provide vestibular-like information, as discussed in the results. The projection to the nMLF provides a direct link to the motor system, since this nucleus has a direct output to the spinal motor neurons. The implication of this nucleus in the escape reaction has been discussed above (Results). Interestingly, in mammals this nucleus also receives indirect input from the vestibule and is involved in gaze maintenance through a control of neck muscles. Given that neck appeared relatively late in vertebrate evolution, it seems likely that the mammalian circuit relaying vestibular information to neck motor neurons is a specialized adaptation of an older, more general system that relayed lateral line (and possibly vestibular) information to body motor neurons.

We also observed direct second-order projections to diencephalic parts of the brain, something that had not been reported previously. Most of these projections course through the poorly defined AL path and include only few fibers, but they are reproducibly observed. Some of them may prefigure third-order projections from the torus to diencephalic centers. For example, the main thalamic target of the torus semicircularis is the lateral preglomerular nucleus. This nucleus arises from the migrated part of posterior tuberculum (M2) which is a target of the second-order projection in the embryo. More unexpectedly, the ipsi- and contralateral anterior branches of AL are apposed to two brain regions that will be the major targets of the diencephalic projection from the torus in amphibians. This may indicate that vertebrates share a program for building a general-purpose scaffold for the processing and integration of mechanosensory information. Modulation of this processing network may result from the withdrawal of specific parts of this primitive scaffold, or alternatively, from maintenance at such a low level as to become virtually undetectable in the adult, when other components are massively increased during larval growth.

## Conclusion

We have shown that the second-order PLL projection can be reproducibly visualized in the early zebrafish brain, and that it is highly stereotyped and highly lateralized. It comprises a major branch to the torus semicircularis and a minor branch to the deep layers of the optic tectum as previously described in adult fish. It also comprises a number of branches that have not been reported so far. Some of the new branches are directed to regions that may correspond to targets of the third-order projection in adult fish or amphibians, or to targets of the ascending vestibular projection in fish or in mammals. We propose that the second-order PLL projection in the zebrafish embryo reveals most or all of a general-purpose scaffold from which subsets of elements will be specifically suppressed or reinforced in various groups of vertebrates later in development, accounting for the known differences in connectivity between the major vertebrate groups.

## Materials and methods

### Fish

Zebrafish (*Danio rerio*) were obtained from Singapore through a local company, Antinea (Montpellier, France), and maintained in standard conditions [32]). Embryos were obtained from pairs of adult fish by natural spawning and raised at 28.5°C in tank water. Ages are expressed as days after fertilization (daf). The islet-GFP line [15] was obtained from Dr H Okamoto.

### Injections

Four-day-old zebrafish were fixed overnight at 4°C in 4% paraformaldehyde in phosphate buffered saline (PBS) and kept at 4°C in PBS. Individual fish were mounted on a coverslip in a drop of 0.7% agar in PBS. The coverslip was secured to a slide with a tiny drop of agar and the lateral line nerve was visualized under Nomarski optics using a 40× long-distance water-immersion objective on a fixed-stage Zeiss (Oberkochen, Germany) Axioscop microscope. DiI (Molecular Probes, now part of Invitrogen, Carlsbad, California, USA) was used at 2 mg/ml in dimethylformamide and was applied with a 1.2 mm OD capillary drawn on a Narishige (Tokyo, Japan) electrode puller (two-step pulling to obtain a short tip of about 30 Mohms resistance). Iontophoretic injection into the nerve was driven by a WPI electrometer (Sarasota, Florida, USA). DiO (Invitrogen, Carlsbad, California, USA; 2 mg/ml in dimethylformamide) was pressure-injected using 5 ms pulses from a PicoSpritzer (Intracel, Shepreth, UK). The capillary used for DiO injection was drawn as before but had its tip broken just before injection. Coverslips with injected embryos were transfered to wells and kept in PBS at 4°C overnight. The embryos were then examined for labeling of the projection in the hindbrain, and re-embedded in 0.7% agar on another coverslip, dorsal side up. The coverslip was again secured to a slide with a tiny drop of agar, and the first-order projection was visualized under fluorescence. An electrode pulled as above was inserted laterally and DiI was injected within the synaptic field. The embryo was again kept overnight at 4°C in PBS and examined the next day for second-order projection.

### Microscopy

All examinations were done on a Nikon (Tokyo, Japan) Microphot microscope fitted with a Princeton Instruments (Trenton, New Jersey, USA) Pentamax camera, using mostly a Nikon 20×, 0.5 NA objective (occasionally 10×, 0.3 NA or water-immersion 40×, 0.55 NA objectives). Results were recorded as Z-stacks with steps of 2 to 10 micrometers, depending on the objective. The Princeton Pentamax camera, Uniblitz shutter (Vincent Associates, Rochester, New-York, USA) and ASI (Eugene, Oregon, USA) focusing stage were controlled by the IPLab 3.6 (Scanalytics, now part of Becton Dickinson, Franklin Lakes, New Jersey, USA) software running on an Apple (Cupertino, California, USA) G4 computer. Further processing of the figures (contrast adjustment, superposition of DiI and GFP images) was mostly done with IPLab 3.9 and, to a minor extent (final adjustments), with Adobe Photoshop running on an Apple G5 computer. In some cases (Figures 2, 5a, 7a,b, 8a and 13) the relevant features from consecutive images were combined using Adobe Photoshop. In Figure 1, the red (DiI) and green (islet-GFP) images correspond to different, non-consecutive levels of the Z-stack. The stereo pictures of Figures 6a and 12a,b were obtained by light deconvolution of the original Z-stack using the 'Rapid Deconvolution' program of IPLab, followed by slanted brightest-pixel projection using the '3D Projector' program. Figure 15c was also treated with the 'Rapid Deconvolution' program.

## Competing interests

The author(s) declare that they have no competing interests.

## Authors' contributions

RFM described the various projections reported in this paper. CB assigned them to specific rhombomeres and expanded the database. AG initiated the experiments, drew the conclusions and drafted the manuscript. All authors read and approved the final manuscript.
